# Integration of 3D Printed Flexible Pressure Sensors into Physical Interfaces for Wearable Robots

**DOI:** 10.3390/s21062157

**Published:** 2021-03-19

**Authors:** Kevin Langlois, Ellen Roels, Gabriël Van De Velde, Cláudia Espadinha, Christopher Van Vlerken, Tom Verstraten, Bram Vanderborght, Dirk Lefeber

**Affiliations:** 1 Robotics & Multibody Mechanics Research Group, Department of Mechanical Engineering, Vrije Universiteit Brussel, 1050 Elsene, Belgium; ellen.roels@vub.be (E.R.); gabriel-mathieu.van.de.velde@vub.be (G.V.D.V.); christopher.van.vlerken@ulb.be (C.V.V.); tom.verstraten@vub.be (T.V.); bram.vanderborght@vub.be (B.V.); dirk.lefeber@vub.be (D.L.); 2 imec, 3001 Leuven, Belgium; 3 Department of Physics, Universidade de Lisboa, 1749-016 Lisbon, Portugal; fc46720@alunos.fc.ul.pt; 4 Flanders Make, 3290 Lommel, Belgium

**Keywords:** wearable robotics, safety in HRI, soft sensors

## Abstract

Sensing pressure at the physical interface between the robot and the human has important implications for wearable robots. On the one hand, monitoring pressure distribution can give valuable benefits on the aspects of comfortability and safety of such devices. Additionally, on the other hand, they can be used as a rich sensory input to high level interaction controllers. However, a problem is that the commercial availability of this technology is mostly limited to either low-cost solutions with poor performance or expensive options, limiting the possibilities for iterative designs. As an alternative, in this manuscript we present a three-dimensional (3D) printed flexible capacitive pressure sensor that allows seamless integration for wearable robotic applications. The sensors are manufactured using additive manufacturing techniques, which provides benefits in terms of versatility of design and implementation. In this study, a characterization of the 3D printed sensors in a test-bench is presented after which the sensors are integrated in an upper arm interface. A human-in-the-loop calibration of the sensors is then shown, allowing to estimate the external force and pressure distribution that is acting on the upper arm of seven human subjects while performing a dynamic task. The validation of the method is achieved by means of a collaborative robot for precise force interaction measurements. The results indicate that the proposed sensors are a potential solution for further implementation in human–robot interfaces.

## 1. Introduction

Exoskeletons are designed to assist, augment, or restore human physical functions by physically interacting with them. In that perspective, robotic assistance can be seen as flow of mechanical power to and from the robot to the musculoskeletal system. This power transfer is mediated through mechanical and sensory components, which are the Physical Human Robot interfaces (PHRi).

Because exoskeletons work closely to the human body, safety and comfort is a critical aspect. Nevertheless, the physical interaction between patients and rehabilitation robotics has been associated with a number of adverse events [1]. He et al. published a review on the major and minor injuries that were reported in literature involving the use of lower limb powered exoskeletons [2]. Skin and soft tissue injury is the most frequent type of wound, happening with all devices in the report (Ekso, Rex, Ekso, and Hal), and it is repetitive, occurring to several subjects in the same study. As the authors state, many of the studies were able to prevent injuries by adding padding. In order to define effective injury-preventing designs, it would therefore be interesting to collect information on the sustained wounds, e.g., pressure acting on the soft tissues. However, it is difficult to summarize any pattern of skin and tissue damages, given the information available and the lack of sensors in padded braces that could monitor forces at the physical interface.

Methods are required to assess the physical human–robot interactions in order to avoid these events and promote a safe deployment of collaborative and rehabilitation robots. In that regard, three approaches are reported in literature.

The first approach is to measure the torques that are generated at the joint level to compute the torque transferred to the user through the physical interface. The interaction torque can be used to compute how the loads are transferred to the user by implementing a model of their connection and interaction. This model is notably complex to obtain since it involves interactions between soft tissues and the components of the interface (orthotic shell, straps, padding, etc). Moreover, when straps are used to connect the user to the device, which is the case in most upper limb [3] and lower limb exoskeletons [4], the forces distributed on the strap may compensate each other and not result in a measurable force at the connection point, while effectively loading the user’s skin. This is the case when the belt is fastened and, consequently, applies a preloading pressure to the limb.

A second approach is to directly place a load cell sensor at the attachment level to measure the interaction force [5]. This removes the need of an accurate model of the robot but still requires a model of the interaction between the soft tissues and the interface to compute pressure distribution. Additionally, load cells cannot be used when the interaction between the user’s limb and the robot link is not mediated by a finite number of attachments, but by a distributed area. Pressure distribution can be useful, being directly related with the safety and comfort felt by the user during the robot operation [6,7].

Single point force measurements are the two previous approaches. The third approach is the distributed approach, which allows to measure the pressure applied on the human skin. Because pressure is critical to ensure safety and comfort, studies have included the integration of pressure sensing devices into exoskeletons. Different technologies have been integrated into interfaces. The most common approach is the use of Force Sensitive Resistors (FSR) [8,9,10,11]. FSRs offer the advantage of very thin (polymer film) construction, together with high spatial resolution. However, FSRs are known to suffer from a number of drawbacks relating to the cell-to-cell variability, considerable hysteresis, sensitivity to shear loading, sensing threshold, temperature sensitivity, sensitivity to bending, contact resistance, creep behaviour, and alterations in response properties with prolonged use. Strategies to compensate for some of these effects are reported in literature [12]. Commercial devices that are based on this technology exist, but they offer limited versatility in terms of shape and dimensions of the sensor, which leads researchers to develop customized solutions [13,14]. Others have integrated pneumatic padding for sensing of physical interaction and shown promising results [15,16,17]. Lenzi et al. have proposed pressure sensors that are based on an optoelectronic circuit [18]. The pressure acting on the sensor deviates a light beam, which creates a potential difference. While the two latter solutions offer promising results in terms of accuracy and linearity of the sensor output, an alternative is proposed with a key benefit.

The additive manufacturing research field has developed techniques for the development of soft capacitive pressure sensors [19,20,21]. To the authors’ knowledge, none of these sensors have been integrated into an exoskeleton. The key benefit of three-dimensional (3D) printed sensors in the field of wearable robots is the opportunity to customize the solution to the application and the individual [22].

**Figure 1:**
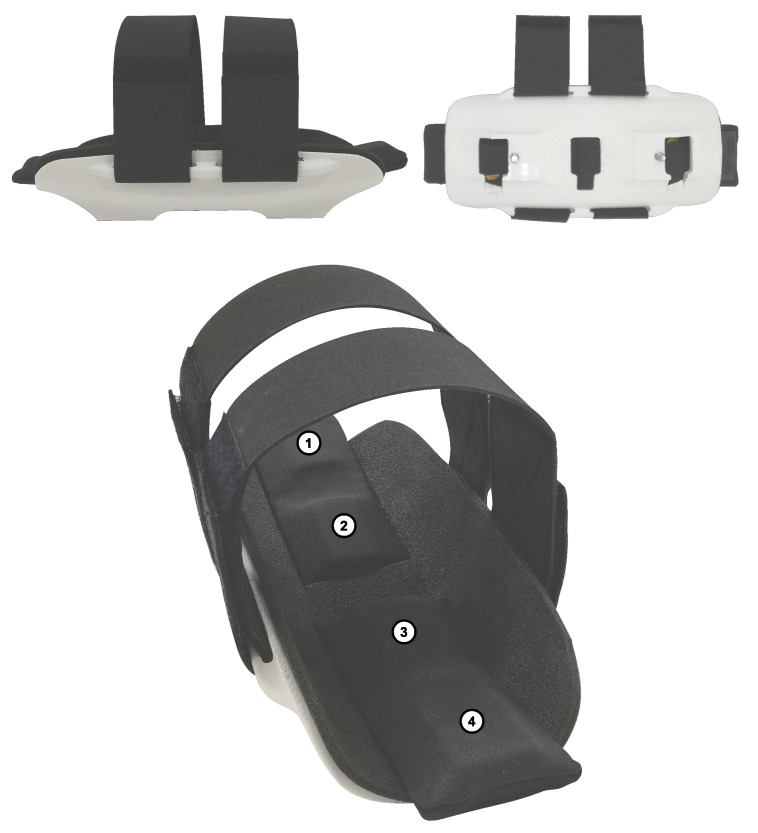
The sensorized shell consists of a three-dimensional (3D) printed shell with four pressure sensors indicated by the numbers 1 to 4.

In this manuscriptm we demonstrate a novel interface for a wearable robot with embedded flexible pressure sensors. We demonstrated it here specifically for an upper arm interface, as shown in Figure 1, but the concept can also be expanded to other applications. The presented pressure sensors are fabricated using flexible conductive filaments printed with commercially available 3D printers. Hereby, the goal is to allow for seamless integration of sensing solutions to physical interfaces of wearable robots. Similarly to [20,21], the concept of the capacitive pressure sensor relies on a parallel plate structure. The read-out electronics that are proposed in this manuscript are cost-efficient and they will allow other researchers to reproduce the results that are presented here. The sensor response is characterized in a test-bench under different loads and conditions. Additionally, a human-in-the-loop calibration is performed using a torque controlled robot, wherein the sensor output is calibrated while the participants are wearing the interface. This is an important step, since it is commonly accepted that biological tissue compliance, curvature, and temperature affect sensor readings [23].

The manuscript is structured, as follows. In the first section of the manuscript, we present the design of the sensor with an emphasis on modelling and the constraints that should be considered in the specific application of an upper arm interface for exoskeletons. Following, the fabrication process is detailed as well as the reading-circuit for the capacitive sensors. After which, in Section 2.5, the characterization method and the results of the sensor in a test-bench are detailed. Subsequently, Section 2.6 presents the method of the human-in-the-loop calibration of the sensor, i.e., while worn by human subjects. All of the results of the test-bench experiments and the human-in-the-loop calibration are discussed in Section 4, and the conclusions are presented in Section 5.

## 2. Methods

### 2.1. Design of Flexible Capacitive Pressure Sensor

**Figure 2:**
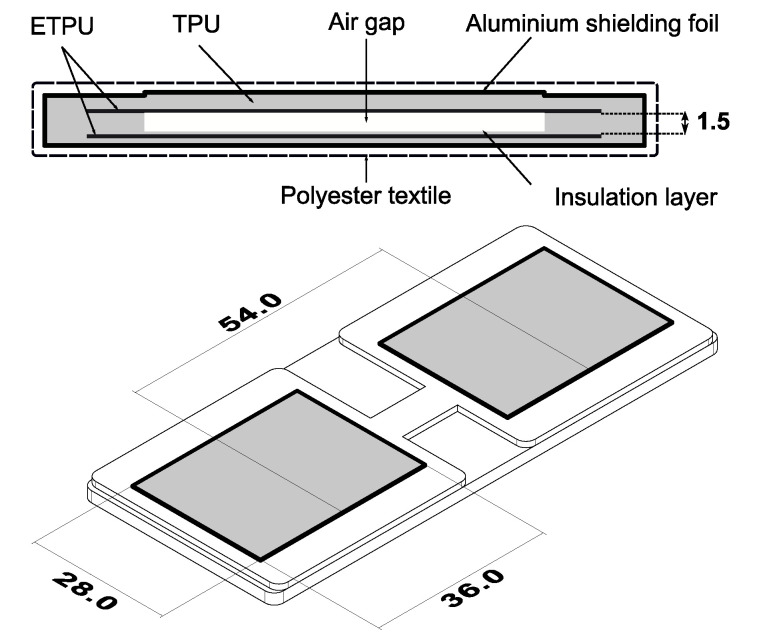
The flexible pressure sensors described in this work measure the change of capacitance in between two flexible conductors (ETPU). The change in capacitance arises due to the fact that the material is flexible and, therefore, will be easily deformed when a force is applied.

**Figure 3:**
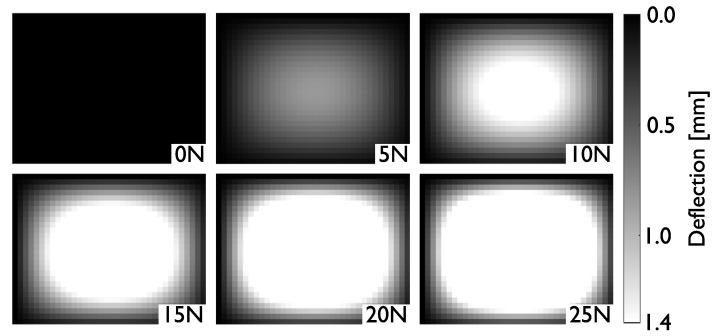
Finite element analysis of the pressure sensor for typical loads inside the interface. Note the stagnating deflection for loads above 10 N.

With our focus being the application of distributed force sensing to the monitoring of human–robot interaction at the upper-arm level, we developed a distributed soft force sensor that is based upon previous work of Schouten et al. [20]. The pressure sensors that are described in this work measure the change of capacitance in between two flexible conductors, see Figure 2. The change in capacitance arises due to the fact that the material is flexible and therefore will be easily deformed when a force is applied. Because the distance between the plates is much smaller than the dimensions of the electrodes (36 mm × 28 mm versus 1.5 mm), the capacitance can be calculated using the parallel plate approximation:

(1) $$C=\frac{\epsilon_{r}\epsilon_{0}A}{d}$$

with *A* being the area of the overlap between the plates, *d* being the distance between the plates, $\epsilon_{r}$ being the relative permittivity of air, and $\epsilon_{0}$ being the permittivity of vacuum. An air gap was introduced between the plates in order to increase the deflection, which, in turn, increases the change in capacitance for a given load, as shown in Figure 2. The deflection of such a plate is modelled using Kirchhoff’s theory of deflection for thin rectangular plates in Matlab^®^. Figure 3 shows the outcomes of the deflection equations, which were calculated with the dimensions shown in Figure 2 with a Young’s modulus of 26 MPa and a Poisson’s ratio of 0.5. The maximal deflection is when the two plates are pressed against each other and only separated by an insulation layer of 0.1 mm.

The deflection of the plate is combined with Equation (1) to calculate the total capacitance of the sensor in the following manner:

(2) $$C_{T}=\epsilon_{r}\epsilon_{0}\int_{A}\frac{dA}{d}$$

The dimensions of the sensor were based on the application and the following constraints that it imposes:

- Number of sensing elements.
  A complex aspect of capacitive pressure sensor design is the read-out electronic circuit. As detailed in Section 2.3, the complexity of the read-out circuit was reduced to a single microcontroller with a minimal spatial footprint, which is easy to integrate and merge to iterative designs. However, the presented simplified read-out electronics result in a limit of four sensing elements. Section 2.3 details the read-out electronics and Section 4 discusses the limitations.
- Area of the sensor.
  The sensor is to be integrated in a cuff, as shown in Figure 1. The objective is to maximize the sensing area and avoid losing information by having the soft tissues interact with non-sensorized area. A smaller sensor increases the spatial resolution of the measurement, but increases read-out complexity. On the other hand, a large sensor will inevitably have to bend to fit the interface, which changes the response of the sensor. Additionally, increasing the area can lead to varying load distribution on the sensor plate and, thus, result in unpredictable response. To fit the specific cuff for this application, the width is fixed to 28 mm to 36 mm. To limit the scope of the study, the sensing elements are placed along the center-line of the interface, which means that pressure distribution is only measured along the length of the arm.
- Desired force range measurement.
  The results from studies on pain pressure thresholds indicate that circumferential compression becomes painful at ∼20–27 kPa [6]. Therefore, it is our objective to develop a sensor that can reliably measure in the range 0–25 kPa. If we consider a sensing area of 10 cm${}^{2}$, then the maximal force to measure is 25 N.

**Figure 4:**
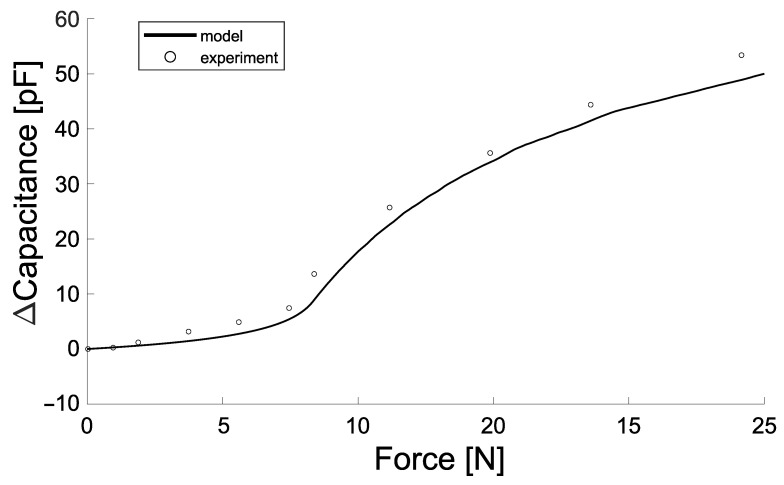
The dimensions of the sensor are determined based on a theoretical model of the sensor. The region of interest for the application is to measure the forces ranging from 0 to 25 N. In this range the model is able to accurately predict the response of the sensor. The capacitance response of the sensor is based on the deflection of the plates which is modelled using a finite element analysis. In Section 2.5, the methods on how these data are obtained are detailed.

The aforementioned constraints lead to the design parameters that are shown in Figure 2. The output of Equation (2) for the presented parameters is compared to experimental data and shown in Figure 4. In this initial experiment, the sensor pad is loaded until 25 N at a compression rate of 0.45$\frac{\mathrm{mm}}{s}$. Section 2.5 details the methods on how this data are captured.

### 2.2. Fabrication

**Figure 5:**
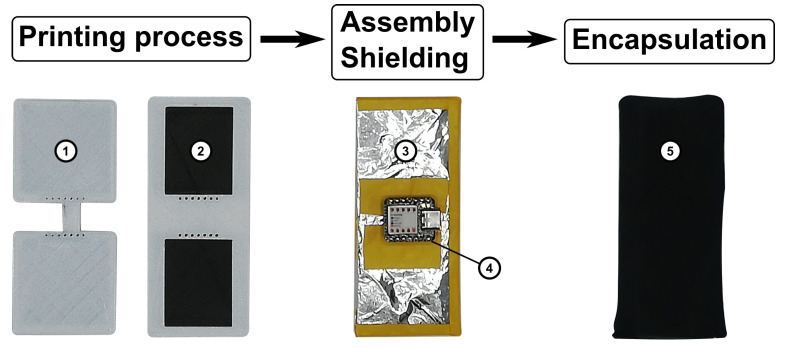
The sensor is composed of two flexible thermoplastic polyurethane materials; a non-conductive TPU filament (1) is used to print the substrate and encapsulant, and a conductive TPU filament (2) is used to print the capacitive plates. The sensor is assembled, shielded (3), and encapsulated in a stretchable fabric (5) to be implemented in a physical interface. A microcontroller unit (4) is integrated to measure capacitance.

A Fused-Fabrication-Filament (FFF) printer (Toolchanger, E3D, Oxfordshire, UK) was used to fabricate the sensor. The sensor is composed of two flexible thermoplastic polyurethane materials; a non-conductive filament (TPU-95A, Ultimaker, Utrecht, The Netherlands) is used to print the substrate and encapsulant, and a conductive filament (PI ETPU, Palmiga Innovation AB, Jonstorp, Sweden) is used to print the capacitive plates. The fabrication steps are depicted in Figure 5. The sensor consists of two separately printed sides. After the completion of the printing process, both sides are joined using double-sided tape. Printing the two sides separately eliminates the need of support material to create the air gap. The air gap creates a more flexible structure and, thus, a more sensitive pressure sensor. Stray capacitances are an important problem when considering wearable electronics. The entire sensor is shielded in an aluminium foil to cope with stray capacitance. The foil is folded around the plates, and a cavity is cut in the foil to house a microcontroller unit (MCU). The folded foil is joined with electrical tape. A commercially available MCU (Seeeduino Xiao, Seeed, Shenzhen, China) is placed as close to the sensing areas as possible, to limit electromagnetic interference. The entire composition is encapsulated in a stretchable polyester fabric to isolate the sensor and achieve comfortable contact with the skin.

### 2.3. Read-Out Electronics

The study is composed of two separate methods. First, a characterization process is performed in a test-bench. This process is performed to analyze the response of the sensor under different loading conditions. However, once such a sensor is placed inside an interface, many factors will influence its response. These are discussed in Section 2.5. Therefore, in the second method, a human-in-the-loop calibration method (i.e., while wearing the interface) is introduced in Section 2.6.

For the test-bench characterization, the capacitance is measured with an LCR-meter. For the human-in-the-loop calibration, to make the sensorized interface portable, the capacitance is measured with a MCU. In [20], it was shown how an electronic circuit and a MCU can achieve accurate capacitance readings. Additionally, in [24], a strategy is explained to measure capacitance using a single MCU. This exact same strategy was implemented here. In short, capacitance can be measured with a MCU by measuring the charging-and-recharging cycle time of the capacitors. A reference capacitor on the MCU maps the charging time to actual capacitance values. The MCU is Cortex M0 (SAMD21G18)-based and it is commercially available on a PCB with a small form factor. The sensorized interface is comprised of four pressure sensors, for which two MCU’s are implemented, one per pair, as shown below. Each microcontroller communicates through a serial communication protocol with a PC running Matlab®. In this manner, a sampling rate of 12 Hz is achieved.

### 2.4. Interface Implementation

Once characterized, the sensors are integrated to the interface, as previously shown in Figure 1. The interface consists of a rigid shell, which is 3D printed in polylactic acid (PLA). The sensors are joined to the inside of the interface which integrates a housing for the MCU and clearance for cabling. The unsensorized areas of the shell are padded with a low density polyethylene foam (LD33). The dimensions of the shell were defined, such that it would fit a wide range of participants. The shell has integrated buckles to fit elastic straps with hook-and-loop fasteners. Four pressure sensors were integrated along the center-line of the shell in a symmetrical disposition. Two sensors were placed on the edges of the interface, which are expected to be the regions with the highest pressure values, and two at the center of the shell. When placed inside the shell, the sensors are slightly bent, due to the curvature radius of the shell. The position of the sensors is such that virtually all of the interacting surface between the arm and the cuff is sensorized, i.e., all contact forces between the interface and the participant are measured by the sensors.

### 2.5. Test-Bench Characterization

Before integrating the sensors in the interface and perform human-in-the-loop calibration, the sensor is characterized in a custom-made test-bench. The test-bench is composed of a linear actuator that consists of a ball screw and a stepper motor onto which a loadcell (LSB200, Futek, Irvine, CA, USA) and an indenter are coupled. The conductive plates of the sensor are connected to the Kelvin clamps of an LCR-meter (E4980AL, Keysight, Santa Rosa, CA, USA). A Raspberry Pi controls the force that is applied onto the pressure sensor and collects all of the measurements of forces and capacitances during the experiment.

The initial experiment to validate the theoretical model described in Section 2.1 is performed in the presented test-bench. The sensor was printed and loaded once, with a linearly varying force ranging from 0 to 25 N at a compression rate of 0.45$\frac{\mathrm{mm}}{s}$. The sensor is only loaded once to reduce the Mullins effect, i.e., the dependency of the response of rubbers on the maximum loading previously encountered. The following experiments were subsequently undertaken;

(a) Step input: foam vs. no-foam.
  A static experiment is performed to define the effects of adding foam on top of the sensor. Foam is placed on top of the sensor for two reasons. First, it distributes the loads more evenly on the capacitive plate. Second, it ensures that the soft tissues interact with the interface through sensorized areas. If the sensors are too flat, then the soft tissues can bend around the sensor and interact with non sensorized areas. This results in a reduced amplitude of the sensor reading and a loss of information. The deformation mechanisms of this foam, such as densification [25], affect the response of the sensor. Three static loads (1 kg, 2 kg, and 3 kg) are placed on the sensor. Two conditions are assessed: no foam vs. low density (LD33) polyethylene foam (3 mm thickness). The loads are placed onto the sensor in a step-like fashion.
(b) Frequency dependence.
  A cyclic experiment to assess the dependency of the sensor response to three different input frequencies (0.1 Hz, 1 Hz, and 2 Hz) in the range of interest for the considered application (10–25 N) during 10 cycles each.
(c) Inter-sensor variability.
  A cyclic experiment to assess the variability across the four sensors that are integrated in the interface. The four sensors were printed with identical printing parameters and, on the same day, using the same filaments, on the same printer.
(d) Cyclic high load input.
  A cyclic experiment to explore the wide sensing range of the sensor, the hysteresis that is exhibited and the drift in a dynamic setup. The cyclic load is applied at a rate of 0.1 Hz at a maximal amplitude of 85 N during 200 cycles.
(e) Indentation location.
  A spherical indenter with a diameter of 10 mm is placed at three different locations of the sensor pad: the center, the bottom extremity (13 mm from the center), and the side extremity (9 mm from the center). A cyclic load is applied at a rate of 0.1 Hz at a maximal amplitude of 55 N during 15 cycles.
(f) Repeatability.
  A single sensor is repeatedly loaded with an identical load. The sensor is loaded at an amplitude of 10 N during 30 s, after which the sensor is left untouched for 90 s. The sequence is repeated four times.

Section 3 reports the results of these experiments.

### 2.6. Human-in-the-Loop Calibration

An experiment is presented, where seven human subjects are performing interactions with the sensorized interface shown previously. The interface is coupled to a torque controlled robot (Franka Panda, Franka Emika). The cobot has seven degrees-of-freedom with a torque sensor in each joint allowing for force and torque to be measured in all directions at the end-effector. The cobot also allows forces to be generated at the end-effector by controlling joint positions and torques. This allows for a direct comparison between the forces that were measured at the end-effector and the capacitance measurements of the sensors.

**Figure 6:**
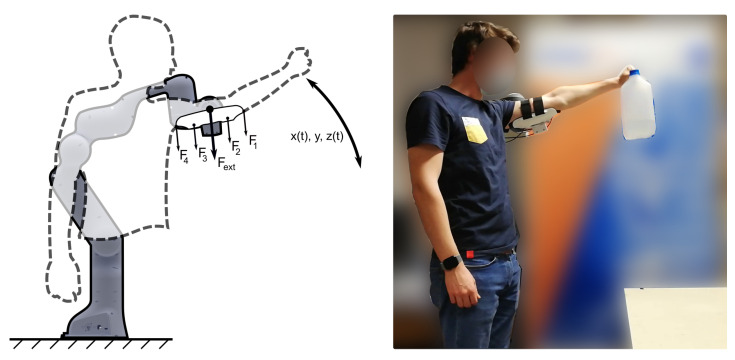
An experiment is designed to calibrate the sensors while the participants are wearing the sensorized interface. Participants are asked to lift a jerrycan up and down, at a self selected speed. The pressure readings are compared to the external forces acting on the end-effector to calibrate the sensors. $F_{1}$, $F_{2}$, $F_{3}$ and $F_{4}$ are the forces measured by the four sensors, $F_{ext}$ is the force measured at the end-effector, x(t), y and z(t) are the spatial coordinates of the end-effector. The motion of the robot is constrained to the xz-plane, such that y remains constant.

The sum of all pressure readings of the sensors should equal the external forces measured at the end-effector, as illustrated in Figure 6. This is true if we assume that the entire area of the soft tissues are interacting through the sensors, i.e., that no information is lost. The area over which the arm interacts with the sensor is assumed to be constant and equal to the sensing area for all sensors. Hereby, pressure measurements can be converted to force measurements. To validate this, the following experimental protocol is proposed.

The hypothesis of this experiment is to prove there is a set of parameters that we can assign to the output of the sensors for which a linear relationship exists between the sum of the forces measured by the pressure sensors and the force measured at the end-effector. This set of parameters should give accurate results, regardless of the anatomy of participants and regardless of the position of the arm relative to the interface, such that

(3) $$F_{ext}=a\left(\sum_{i=1}^{4}F_{i}\right)+b$$

with $F_{ext}$ being the force measured at the end-effector, $F_{i}$ being the individual force measurements of the four pressure sensors, *a* being a dimensionless conversion unit, and *b* being the pre-load of the interface to the arm in *N*. This pre-load is determined at the start of the experiment during the static calibration procedure.

Seven subjects are asked to don the sensorized cuff (which is coupled to the cobot) and are asked to do a lifting task. The lifting task comprises a table onto which a jerrycan with a mass of 1.5 kg, which is initially placed on a table. The participant is asked to lift the can above his/her head, up to an indicated level (as indicated by means of a line drawn onto a panel) after which they can lower the load back onto the table. This lifting motion is repeated in a loop until one minute has passed. The speed at which the load is lifted is self-selected by the subject. Before the experiment, a static calibration procedure to measure the initial strapping pressure or pre-load is performed. The participant is asked to don the interface and stay immobilized for ten seconds. The pressure readings obtained during that time are averaged and this values is subsequently subtracted from the future readings. The subjects can adjust the tension in the straps before the start of the experiment to ensure a comfortable fit. The tension in the straps is not adjusted at any time during the experiment. Markings on the ground ensure that the participants’ resting position relative to the robot remains similar across the users. Consent to the experiment was given prior to the experiment by all participants. All of the human subject studies were approved by the Ethical Commission of the UZ Brussel (B.U.N.143201942279), and the participants gave informed written consent prior to participation.

The cobot is programmed to provide a force assisting the lifting motion in a similar fashion to how a passive upper-body exoskeleton would. This is achieved by means of a joint impedance control with the following parameters expressed in $\frac{Nm}{rad}$;

(4) $$k_{1}=3000;k_{2}=30;k_{3}=3000;k_{4}=30;k_{5}=3000;k_{6}=3000;k_{7}=0.01;$$

This joint impedance control results in the end-effector of the robot being constrained in the xz-plane, as shown in Figure 6. The initial position of the robot is set at the highest lifting position of the subject. As such, the user only experiences forces that are directing upwards, i.e., a force that assists the user in lifting the arm and provides resistance when lowering the arms. This force is proportional to the angle of the robot joints, where the highest force is exerted at the lowest arm position and the lowest force at the highest arm position. This force is different for each subject, since the initial position is adjusted for the height of the subject.

The position of the end-effector and the forces acting on it are recorded at 1 kHz by the supplied electronics of the robot. The pressure readings are recorded on a separate computer at an average sampling rate of 12 Hz. For each subject, three trials are performed, from which only the third is recorded. This is to compensate for the densification effects that are induced by the foam and polyurethane of the sensors.

The outcome in force and position of the end-effector for each subject are shown in the section below.

## 3. Results

### 3.1. Results Test-Bench Characterization

#### 3.1.1. Effect of Foam

**Figure 7:**
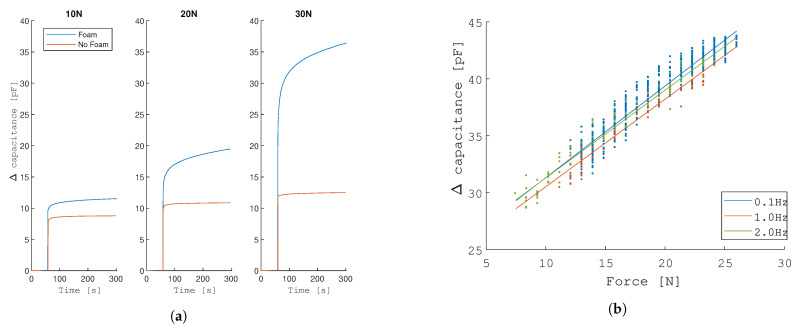
Test-bench characterization results (A): Adding foam on top of the sensor affects the response of the sensor significantly, since the load is more evenly distributed on the plate. No strong frequency-dependency of the response is observed. (**a**) Effects of adding foam on top of the sensor to improve uniformity of applied loads. Three loads (1 kg, 2 kg and 3 kg) are placed onto the sensor. (**b**) Effects of adjusting the frequency of the load.

There are two effects mainly observed in Figure 7a. The first effect is that the addition of polyethylene foam results in a higher capacitance change across the loads. The difference in capacitance between the 10 N static load and the 30 N static load results in a change of capacitance of 24.8 pF, as compared to only 3.7 pF without foam. This is due to the fact that the foam distributes the load more evenly. Because the indenter is a rigid plate, with the same area as the conductive plate, when a load is applied, the upper plate of the sensor is more loaded onto the sides of the plates, effectively loading the top plate in shear. This results in smaller deflections of the top plate of the sensor. A second observation is that the dynamics of the foam play an important role in the deflection of the plates. The response time of the sensor is significantly longer due to the creep behaviour of the foam. The computed response time is 0.58 s in the no-foam condition as compared to 1.26 s for the foam condition, for a load of 10 N.

#### 3.1.2. Effect of Frequency

The sensor is assessed under three periodic loads at frequencies of 0.1 Hz, 1 Hz, and 2 Hz, with a force amplitude ranging from 10–25 N, which is assumed to be the region of interest. The results that are shown in Figure 7b are the first 10 cycles. No strong frequency-dependency of the response is observed.

#### 3.1.3. Inter-Sensor Variability

**Figure 8:**
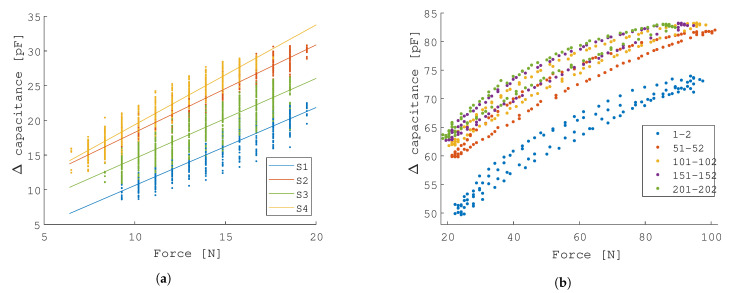
Test-bench characterization results (B): the four printed sensors exhibit negligible differences in response. On cyclic experiment, the drift of the sensor can be observed. After 100 cycles, the drift settles. (**a**) Four pressure sensor were printed and assessed. A cyclic load at a frequency of 0.1 Hz is applied. (**b**) A cyclic experiment at 0.1 Hz to explore the wide sensing range of the sensor, the hysteresis that is exhibited, and the drift in a dynamic setup.

Four sensors are loaded under the same conditions, i.e., a periodic load of 0.1 Hz in the force range that is of interest for the application. The four sensors exhibit negligible differences in response, although an offset in capacitance is observed in Figure 8a. These offset differences are likely due to the fact that filament properties can vary significantly.

#### 3.1.4. Wide Range Loading, Hysteresis & Dynamic Drift

The sensor is loaded under a wide force range at a frequency of 0.1 Hz and it exhibits significant drift, as seen in Figure 8b. Interestingly, in the first 50 cycles, an important drift can be observed, which settles by the 100th cycle. This drift is directly related to the results that are shown in Figure 7a, and it is due to the creep behaviour of the foam. This creep behaviour results in drift of the sensor and imposes limitations in terms of pressure reading. The hysteresis error is 15.56% and calculated considering the maximum difference in sensor output during loading and unloading over all cycles and expressed as a percentage of the working range, as indicated in Equation (5)

(5) $$\mathrm{Hysteresis}\;\mathrm{error}=max\frac{F_{measured,load}-F_{measured,unload}}{F_{measured,max}}\cdot 100$$

#### 3.1.5. Location of Indentation

**Figure 9:**
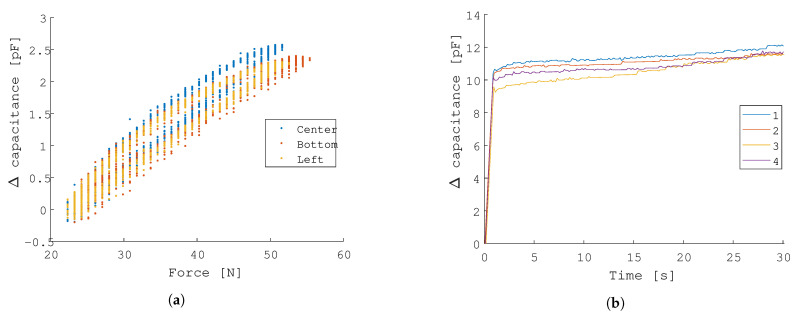
Test-bench characterization results (C): the response of the sensor when subjected to loads at different locations and the repeatability of the sensor is assessed. (**a**) Effects of indenting the sensor at three different locations (center, bottom, and left). (**b**) Repeatability of a single sensor under a constant load of 10 N.

A single sensor pad is indented with a spherical indenter at three different locations: the center of the pad, the bottom extremity (13 mm from the center), and the side extremity (9 mm from the center). A difference in response is measured, at peak force the absolute capacitance is measured to be 2.57 pF, 2.35 pF, and 2.30 pF, respectively, as shown in Figure 9a.

#### 3.1.6. Repeatability

The maximal difference across the four trials is 1.07 pF, computed at the 1 s mark shown in Figure 9b.

### 3.2. Results: Human-in-the-Loop Calibration

#### 3.2.1. Position and Forces of Robot

**Figure 10:**
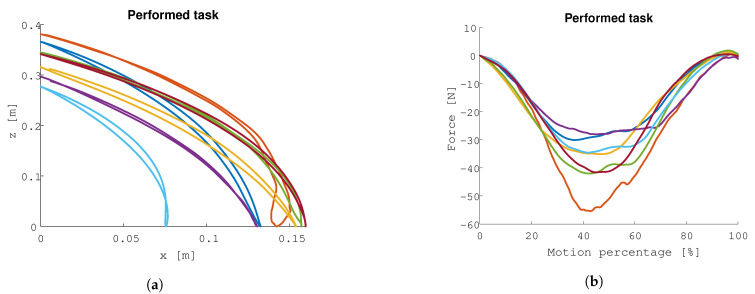
Human-in-the-loop calibration results: motion and forces measured at the end-effector. The trajectories and forces vary significantly across subjects. (**a**) The average motion for each participant of the end-effector of the robot during the experiment. (**b**) Average force for each participant of the end-effector of the robot during the experiment.

Each participant repeated the lifting task during 60 s. The motion trajectories that are shown in Figure 10a are the average trajectories across all repetitions. Each participant performs a unique trajectory, with clear differences that can be explained by the arm length and height of the participant. The differences in trajectory result in different forces acting on the participants, since the robot is programmed to perform joint impedances. The highest average force across all repetitions attains 55 N.

#### 3.2.2. Prediction Accuracy

**Figure 11:**
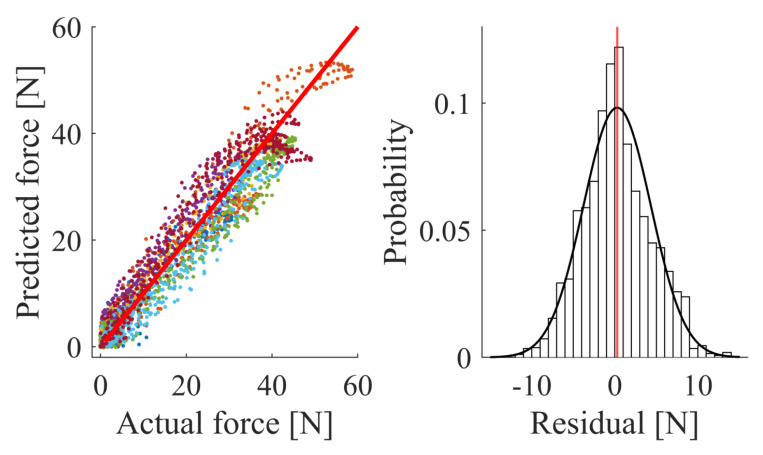
The calibration of the sensors results in a predicted force that matches the actual force with *R*^2^-value of 90.97% across all of the participants. The standard deviation of the residuals between the predicted force and the actual force is 4.1 N.

Firstly, in Figure 11, the prediction accuracy is presented, where the ordinate is the total force estimated by the pressure sensors, as per Equation (3), and the abscissa is the actual force that is measured by the cobot. The y=x line indicates the ideal case where the estimation equals the actual measurement. The $R^{2}$-value is calculated to be 90.97%. Secondly, the histogram of the prediction errors is shown. The residuals indicate a standard deviation on the measurement of force of 4.1 N.

#### 3.2.3. Pressure Distribution

**Figure 12:**
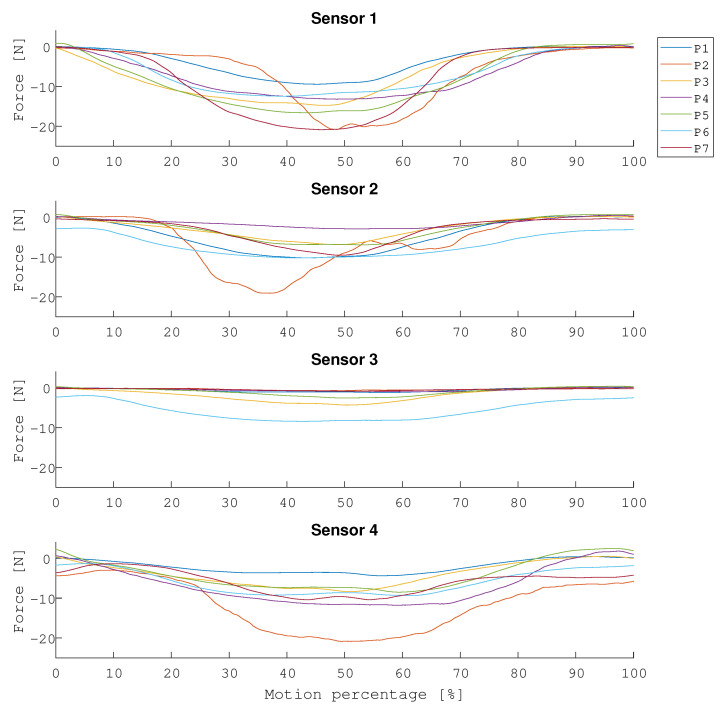
The pressure readings of each pressure sensor during the entire motion is shown. The pressure values that are shown are the average values across all participants.

In Figure 12, the pressure readings of each pressure sensor during the entire motion is shown. The highest pressure across all participants is measured by sensor 1 and sensor 4, which suggests that the arm interacts more with the edges of the interface than with the center, as previously noted by [9].

## 4. Discussion

Pressure sensing at the interface level is known to be an important source of information with regard to the safety and comfort of wearable sensors. The geometry and compliance of physical interfaces between humans and machines can be based on data from pressure measurements, resulting in so-called data-driven designs. However, integrating pressure sensors into these systems is costly if achieved through commercial sensory apparatuses. Moreover, commercial sensors limit the potential for iterative or generative designs. On the other hand, low-cost solutions exist, but integration is complex and the response can be difficult to model and predict. The calibration of these systems is usually performed on test-benches, which do not always accurately represent the interaction with soft tissues. Perhaps this is one of the major reasons why we have not seen the integration of more pressure sensing systems in exoskeletons. In this manuscript, we propose the design of such a low-cost solution and show how human-in-the-loop calibration of the sensor can be performed by using a robotic manipulator. The sensors were characterized in a test-bench a priori to assess the repeatability of the sensors and to assess the range of forces and frequencies that can potentially deliver conclusive data. However, in this characterization process it was shown how changing the properties of the interaction material (i.e., foam) changes the response of the sensor. The dynamics of the foam have to be taken into account when using such sensors. By extension, the soft tissues interacting with the sensors are going to influence the response; therefore, as noted before in [23], it is imperative for the sake of correct calibration to emulate the interaction with soft tissues as closely as possible, or better yet, perform a human-in-the-loop calibration.

No strong frequency-dependency of the response is observed across the ranges of frequencies that were analyzed. Because participants are performing a periodic task, i.e., lifting a mass up and down, the impact of the drift that is shown in Figure 7a is less of a problem than if the participants were to take breaks or perform experiments on a much longer time scale. In that case, strategies to limit the effects of drift and long response time can be undertaken. In that endeavour works such as [12] can be a source of inspiration.

However, the important aspect that is illustrated with this experiment is that current collaborative robot technology allows for human-in-the-loop calibration processes that can be beneficial to cope with the complexity of modelling soft tissue behaviour. Even though the mechanical properties soft tissue is an active field of research, it is mostly focussed on medical and surgical aspects, which is not directly applicable to exoskeletons and wearable robots in general [6]. Additionally, the differences across individuals are significant, and they have been shown to be related to age, sex, anatomical location [26], as well as skin condition such as the grade of scarring [27]. This leads to the idea that a general model can only be of limited use and, thus, an individual or customized process might be inevitable for applications where optimal comfort and/or safety is desired.

On a related aspect, safe and comfortable pressure ranges that can be applied onto humans are yet to be defined [8]. An important obstacle in that regard is the fact that scientist do not have the tools to quantify and document issues related to discomfort. In [17], a method is described that demonstrated the effects of applied pressure on comfort perceived by users wearing an upper arm interface. Skin related issues are often considered minor during clinical issues, since they can often be compensated by adding padding or adjusting the straps [2]. However, a pressure monitoring system would allow for quantifying these issues and providing valuable information to exoskeleton designers to improve the comfort level while wearing these devices. This is important, since the discomfort that is associated with wearing these devices is still a major barrier for a wide adoption of the technology [28].

While the sensor developed in this study provides a potential solution for further investigations, it is limited in the fact that only normal pressure can be measured. This is important, since shear forces have been found to have a larger influence on e.g., pressure ulcers than normal forces [29]. Especially since it it was shown that a major part of people who experienced a spinal cord injury cope with pressure injuries during their lifetime with dramatic changes in their skin structures that are likely to break down with a minimal amount of shear [30].

In terms of performance, we found similar results to the ones that were reported in [15] where the difference between measurements with the pressure sensor and the loadcell are ranging from 4.31 to 9.63% across three users. In [31] an absolute error between 0.84 to 2.83 N per sensing pad is reported for four subjects. This was obtained with static loads with amplitudes going up to 150 N.

Additive manufacturing is one of the enabling technologies that not only allows for customization of products, but also simplifies the process of integrating the sensors in a seamless manner, e.g., by integrating the signal cables into the structure of the device. In this manuscript, we demonstrated the development of a wearable sensor designed for an upper arm interface. The application imposed specific requirements in terms of dimensions of the sensor, sensing range, and integration. The same method can be applied to develop sensorized interfaces for other body parts, such as the lower limbs or torso. In the case of the lower limbs, the interfaces are subjected to higher forces and, thus, a less compliant sensor design might be required. Perhaps a sensor that is more similar to the design shown in [20]. In other cases, even more compliant designs might be desired, for which materials, such as silicones, could provide more options in terms of stiffness.

The proposed sensors capitalize on recent developments in additive manufacturing. They promote iterative and customized design, such as customized interfaces for exoskeletons [22], which are believed to be an important aspect in the field of exoskeletons.

## 5. Conclusions

A sensorized interface for wearable robot applications was developed. The sensors are 3D printed pressure sensor, which are based on parallel plate capacitors, using flexible and conductive filaments. The response of the sensor was accurately modelled in a pressure range of 0–25 kPa. The sum of the pressure sensor measurements can accurately predict the total force acting on human subjects, with a coefficient of determination of 90.97% and a standard deviation of 4.1 N for the experimented task. The sensor was calibrated with two different methods. First, the sensor is characterized in a test-bench that comprises an automated sensorized indenter. The sensor was assessed in terms of how the response of the sensor is affected by three different phenomena. The first one, adding foam on top of the sensor, to more uniformly distribute the loads, is shown to affect the response time and drift of the sensor. Secondly, the sensor’s response relative to the frequency of the load was shown to have minimal effects for a periodic lifting task. Thirdly, the inter-sensor variability test showed that it is possible to manufacture multiple sensors with sufficient tolerances to perform pressure measurements for exoskeletons. The human-in-the-loop calibration resulted in an accurate estimation by the pressure readings of the external force. In the future, the benefits of using the pressure sensor for high-level controllers will be assessed.

## Data Availability

The data presented in this study are available on request from the corresponding author.
